# Early Cardiac Evaluation, Abnormal Test Results, and Associations with Outcomes in Patients with Acute Brain Injury Admitted to a Neurocritical Care Unit

**DOI:** 10.3390/jcm13092526

**Published:** 2024-04-25

**Authors:** Abhijit V. Lele, Jeffery Liu, Thitikan Kunapaisal, Nophanan Chaikittisilpa, Taniga Kiatchai, Michael K. Meno, Osayd R. Assad, Julie Pham, Christine T. Fong, Andrew M. Walters, Koichiro Nandate, Tumul Chowdhury, Vijay Krishnamoorthy, Monica S. Vavilala, Younghoon Kwon

**Affiliations:** 1 Department of Anesthesiology and Pain Medicine, Harborview Medical Center, University of Washington, Seattle, WA 98104, USA; cfong@uw.edu (C.T.F.); walters3@uw.edu (A.M.W.); knandate@uw.edu (K.N.); vavilala@uw.edu (M.S.V.); 2 Department of Biosciences, Wiess School of Natural Sciences, Rice University, Houston, TX 77005, USA; jl226@rice.edu; 3 Department of Anesthesiology, Faculty of Medicine, Prince of Songkla University, Hat-Yai 90110, Thailand; thitikan070@gmail.com; 4 Department of Anesthesiology, Siriraj Hospital, Mahidol University, Bangkok 73170, Thailand; nophanan.chi@mahidol.edu (N.C.); taniga@gmail.com (T.K.); 5 Department of Medicine, University of Washington, Seattle, WA 98104, USA; meno@uw.edu (M.K.M.); oassad@uw.edu (O.R.A.); pjulie3@uw.edu (J.P.); 6 Department of Anesthesiology and Pain Medicine, Toronto Western Hospital, University of Toronto, Toronto, ON M5S 1A1, Canada; tumulthunder@gmail.com; 7 Department of Anesthesiology, Duke University, Durham, NC 27708, USA; vijay.krishnamoorthy@duke.edu; 8 Department of Cardiology, Harborview Medical Center, University of Washington, Seattle, WA 98104, USA; yhkwon@uw.edu

**Keywords:** cardiac evaluation, neurocritical care, traumatic brain injury, ischemic stroke, subarachnoid hemorrhage, intracerebral hemorrhage, troponin, electrocardiogram, beta-natriuretic peptide

## Abstract

**Background:** to examine factors associated with cardiac evaluation and associations between cardiac test abnormalities and clinical outcomes in patients with acute brain injury (ABI) due to acute ischemic stroke (AIS), spontaneous subarachnoid hemorrhage (SAH), spontaneous intracerebral hemorrhage (sICH), and traumatic brain injury (TBI) requiring neurocritical care. **Methods**: In a cohort of patients ≥18 years, we examined the utilization of electrocardiography (ECG), beta-natriuretic peptide (BNP), cardiac troponin (cTnI), and transthoracic echocardiography (TTE). We investigated the association between cTnI, BNP, sex-adjusted prolonged QTc interval, low ejection fraction (EF < 40%), all-cause mortality, death by neurologic criteria (DNC), transition to comfort measures only (CMO), and hospital discharge to home using univariable and multivariable analysis (adjusted for age, sex, race/ethnicity, insurance carrier, pre-admission cardiac disorder, ABI type, admission Glasgow Coma Scale Score, mechanical ventilation, and intracranial pressure [ICP] monitoring). **Results**: The final sample comprised 11,822 patients: AIS (46.7%), sICH (18.5%), SAH (14.8%), and TBI (20.0%). A total of 63% (*n* = 7472) received cardiac workup, which increased over nine years (*p* < 0.001). A cardiac investigation was associated with increased age, male sex (aOR 1.16 [1.07, 1.27]), non-white ethnicity (aOR), non-commercial insurance (aOR 1.21 [1.09, 1.33]), pre-admission cardiac disorder (aOR 1.21 [1.09, 1.34]), mechanical ventilation (aOR1.78 [1.57, 2.02]) and ICP monitoring (aOR1.68 [1.49, 1.89]). Compared to AIS, sICH (aOR 0.25 [0.22, 0.29]), SAH (aOR 0.36 [0.30, 0.43]), and TBI (aOR 0.19 [0.17, 0.24]) patients were less likely to receive cardiac investigation. Patients with troponin 25th–50th quartile (aOR 1.65 [1.10–2.47]), troponin 50th–75th quartile (aOR 1.79 [1.22–2.63]), troponin >75th quartile (aOR 2.18 [1.49–3.17]), BNP 50th-75th quartile (aOR 2.86 [1.28–6.40]), BNP >75th quartile (aOR 4.54 [2.09–9.85]), prolonged QTc (aOR 3.41 [2.28; 5.30]), and EF < 40% (aOR 2.47 [1.07; 5.14]) were more likely to be DNC. Patients with troponin 50th–75th quartile (aOR 1.77 [1.14–2.73]), troponin >75th quartile (aOR 1.81 [1.18–2.78]), and prolonged QTc (aOR 1.71 [1.39; 2.12]) were more likely to be associated with a transition to CMO. Patients with prolonged QTc (aOR 0.66 [0.58; 0.76]) were less likely to be discharged home. **Conclusions**: This large, single-center study demonstrates low rates of cardiac evaluations in TBI, SAH, and sICH compared to AIS. However, there are strong associations between electrocardiography, biomarkers of cardiac injury and heart failure, and echocardiography findings on clinical outcomes in patients with ABI. Findings need validation in a multicenter cohort.

## 1. Introduction

Patients with acute brain injury (ABI) due to neurological/neurosurgical emergencies are often managed in a neurocritical care unit (NCCU) [1,2]. Severe ABI shares common underlying mechanisms that potentially contribute to the development of cardiovascular abnormalities across this diverse patient population. Shared mechanisms include neurogenic stress, neuroinflammation, and autonomic dysfunction [3,4]. While no prospective, multicenter clinical trials to date have examined the prevalence of cardiac abnormalities after ABI, in the last five years, small-sample, single-center clinical prospective and retrospective studies suggest that cardiac abnormalities such as elevated cardiac troponin, beta-natriuretic peptide, and prolonged QTc interval may be present after ABI and may be associated with poor outcomes [5,6,7,8,9,10,11,12].

Cardiac investigation entails the performance of an electrocardiogram (ECG) and testing for QT prolongation, testing for biomarkers of cardiac injury such as cardiac troponin (cTnI), biomarkers of heart failure such as beta-natriuretic peptide (BNP), and reduced ejection fraction (EF) on a transthoracic echocardiogram (TTE). Mechanistically, all these perturbations can be associated with neurogenic cardiac dysfunction. Previous literature revealed that myocardial injury is a mediated process due to the release of norepinephrine from myocardial sympathetic nerves after spontaneous subarachnoid hemorrhage (SAH), which releases cardiac enzymes (cTnIs) [13]. This process leads to worsening cardiac dysfunction, which presents as left ventricular systolic dysfunction, myocardial infarction, pulmonary congestion, and cardiovascular morbidity. Elevated cTnI levels have been noted in 20–68% of SAH patients with no prior cardiac disease [14,15,16] and in 30% of patients with isolated TBI [17] within 24 h of admission. Cardiovascular abnormalities have also been reported in patients with spontaneous intracerebral hemorrhage (sICH) [18]. Similarly, abnormal TTE may be observed in patients with traumatic brain injury (TBI) [19]. Elevated cTnI levels have been associated with poorer Hunt and Hess grade (HH) and delayed cerebral ischemia, and cTnI elevation may be a risk factor for increased mortality in patients with TBI [20].

However, it is unclear if specific types of ABI, such as acute ischemic stroke (AIS), sICH, SAH, and TBI, receive any cardiac evaluation during their early (<72 h) stay in the NCCU and how patients who receive early cardiac evaluation differ from those who do not receive any cardiac evaluation. In addition, the association between cardiovascular abnormalities in ABI and clinical outcomes at discharge remains an active research area. It is essential to understand who gets assessed to benchmark the prevalence of cardiac evaluation amidst the constantly evolving literature. Similarly, studying more than one ABI type is necessary to understand the nuances and differences between the ABI patient populations. Hence, our study examined (1) the frequency and factors associated with receiving early cardiac evaluation after ABI and (2) the association between cardiovascular abnormalities and clinical outcomes.

## 2. Materials and Methods

### 2.1. Institutional Review Board Approval

This study (STUDY00009382-MOD00016515) received approval from the Institutional Review Board (IRB) at the University of Washington on 20 August 2023, with a waiver of consent.

### 2.2. Study Design, Participants, and Clinical Setting

This retrospective single-center observational study was conducted at Harborview Medical Center, a Level I trauma and Comprehensive Stroke Center serving the Washington, Wyoming, Alaska, Montana, and Idaho (WWAMI) regions. Patients ≥ 18 years of age admitted to the neurocritical care service between 1 January 2014 and 31 December 2022 were screened for eligibility. We included patients with AIS, sICH, SAH, and TBI, as these conditions represent a diverse range of ABIs requiring specialized neurological and neurosurgical critical care. Of note, patients with TBI in this cohort represent those with isolated TBI and not polytrauma. Patients with other admitting diagnoses were excluded from the study to maintain a focused examination of the association between cardiovascular abnormalities and discharge outcomes in the target patient population, as mechanisms of cardiac dysfunction may vary in the non-ABI patient population.

At our hospital, there is no protocol or policy for routine cardiac evaluation in ABI. It is left to the neurocritical care clinicians to order any cardiac evaluation, with no protocolized frequency, except that cTnI levels > 0.04 ng/mL will be trended until they peak. BNP and TTE are not protocolized, and often, point-of-care ultrasounds may be performed at the bedside, which may be used to make clinical decisions such as ordering a TTE performed by the cardiologist. The option to order ECGs, cTnIs, and BNPs is available in the admitting order set but is not pre-selected.

### 2.3. Data Collection

We examined demographic characteristics: age, sex, ABI type, admission Glasgow Coma Scale (GCS) score, mechanical ventilation status, and intracranial pressure (ICP) monitoring. Pre-admission cardiac comorbidity was categorized using the Elixhauser comorbidity index based on International Classification of Diseases versions 9 and 10 [21,22]. We categorized patients into the following age groups: 18–44 years, 45–64 years, 65–79 years, and >80 years, and by their admission GCS into mild (GCS 13–15), moderate (GCS 9–12), and severe (GCS 3–8) groups. We collected the following data on cardiac evaluation: cTnI, BNP, prolongation of QTc interval from ECG, and ejection fraction (EF) from TTE within the first 72 h of admission to the intensive care unit. Discharge outcomes were categorized as all-cause in-hospital mortality, death by neurological criteria (DNC), transition to comfort measures only (CMO), and transition to home.

### 2.4. Definition of Cardiac Abnormalities

Cardiac troponin and beta-natriuretic peptide: Due to the heterogeneity in the reported ranges of cTnI [20,23,24,25,26,27,28,29,30,31,32] and BNP [33,34,35,36] across the ABI types, we elected to categorize the cTnI and BNP values obtained in our cohort into four quartiles as shown in Table 1. Interestingly, the ABI types demonstrated different cut-offs for the median and the quartiles for cTnI (TBI expressed lower cTnI ranges, while SAH demonstrated the highest). At the same time, those for BNP were higher for TBI.

Low ejection fraction (EF) was defined as the EF < 40% on TTE. We abstracted information related to the corrected QT interval, which we categorized as normal or prolonged QTc, using greater than 450 milliseconds in males and greater than 460 milliseconds in females as cut-offs [37].

### 2.5. Statistical Analysis

Descriptive statistics were used to describe the characteristics of the study sample. To examine the trends in any cardiac evaluation over the study period, we used 2014 as the reference year and reported unadjusted odds ratios for cardiac evaluations for each subsequent year. To examine the trends in the frequency of cardiac evaluations by admitting diagnosis over the study period, we performed a Cochrane-Armitage Trend Test. To investigate factors associated with the performance of cardiac evaluation as well as specific cardiac tests, we adjusted for age, sex, race/ethnicity, insurance carrier, pre-admission cardiac disorder, ABI type, admission GCS, mechanical ventilation, and intracranial pressure (ICP) monitoring, and we used AIS as the reference group. To examine if cTnI, BNP, prolonged QTc, and EF < 40% were associated with the discharge outcomes, we adjusted for age, sex, race/ethnicity, insurance carrier, pre-admission cardiac disorder, ABI type, admission GCS, mechanical ventilation, and ICP monitoring. For cTnI and BNP, we considered the <25th quartile group as the reference group. Factors on univariate analysis with *p* < 0.05 were selected for multivariable analysis. Results were expressed as adjusted odds ratios (aOR) and 95% confidence intervals (CI). We also explored the relationship between cTnI, BNP, and EF < 40%. We performed univariable analysis between cTnI, BNP, and EF <40%, using <25th quartile as the reference group. A Bonferroni-corrected [38] *p*-value of <0.05 was considered statistically significant. Statistical analysis was conducted using RStudio version 2023.06.0 [39].

## 3. Results

### 3.1. Sample Characteristics

The final sample comprised 11,822 patients: AIS (46.7%, *n* = 3488), sICH (18.5%, *n* = 1383), SAH (14.8%, *n* = 1103), and TBI (20.0%, *n* = 1498); males (58.8%, *n* = 4393); non-white (24.6%, *n* = 1840).

### 3.2. Trends in Cardiac Evaluation over the Study Period

**Figure 1:**
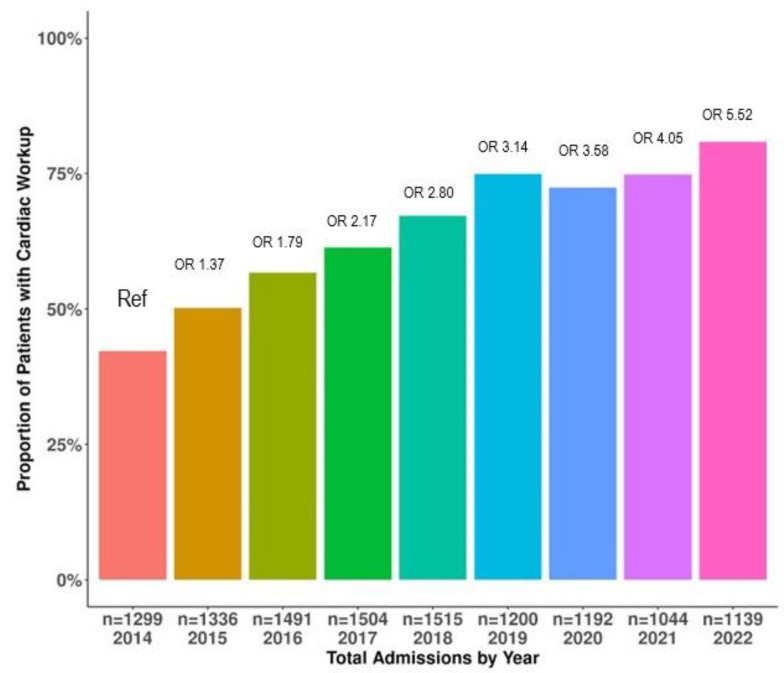
The increase in the proportion of patients with ABI receiving at least one cardiac evaluation from 2014 to 2022. Abbreviations: OR: odds ratio. Note: 2014 = ref = reference year; all subsequent years have unadjusted odds ratios (OR) with *p* < 0.05.

Figure 1 shows the increase in the proportion of patients with ABI receiving at least one cardiac evaluation from 2014 to 2022. The results of the trend analysis indicate that the proportion of patients receiving any cardiac evaluation was higher in 2022 (80%) when compared to 2014 (42%), which was our reference year of admission (*p* < 0.0001).

Compared to 2014, the cardiac evaluations were statistically significantly higher in the subsequent years (2015: odds ratio, OR 1.37 [1.31; 1.77], 2016: OR 1.79 [1.67; 2.26], 2017: OR 2.17 [2.11; 2.89], 2018: OR 2.80 [2.76; 3.78], 2019: 3.14 [2.91; 4.05], 2020: 3.58 [3.39; 4.77], 2021: OR 4.05 [3.49; 4.99], and 2022: OR 5.52 [4.47; 6.44].

### 3.3. Figure 2 Demonstrates the Trends in the Type of Cardiac Evaluation amongst ABI Types over the Study Period

Overall, we observed an increase in individual types of cardiac evaluation for ECG (64.8% in 2014, 72.1% in 2022), BNP (5.6% in 2014, 50.8% in 2022), and cTnI (11.5% in 2014, 64.8% in 2022), but not for TTE (59.9% in 2014, 55% in 2022).

**Figure 2:**
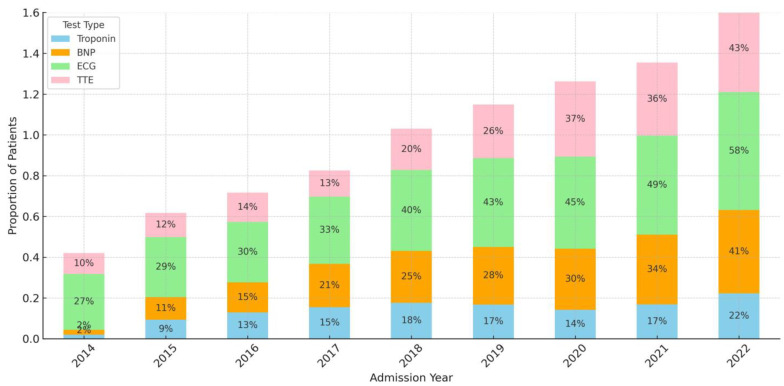
Trends in the type of cardiac evaluations over the study period. Abbreviations: ECG: electrocardiogram; BNP: beta-natriuretic peptide; TTE: transthoracic echocardiogram.

### 3.4. Trends in Specific Type of Cardiac Testing Stratified by ABI Type

**Figure 3:**
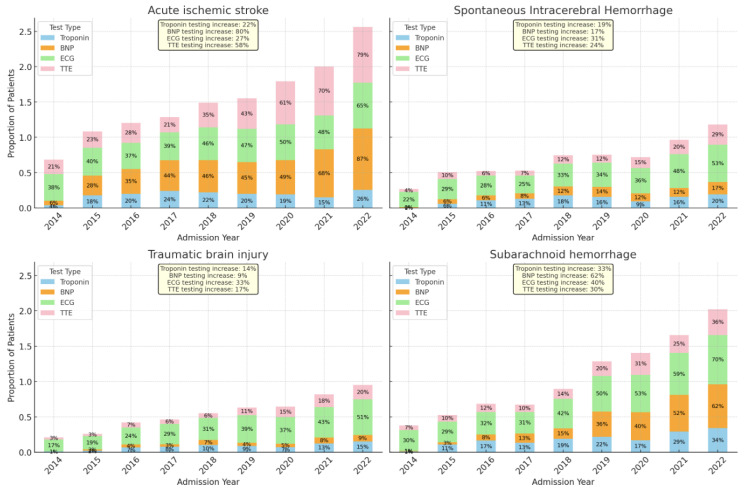
Trends in the type of cardiac evaluation amongst the different acute brain injury groups. Abbreviations: ECG: electrocardiogram; BNP: beta-natriuretic peptide; TTE: transthoracic echocardiogram.

Figure 3 Demonstrates Trends in the Specific Type of Cardiac Testing Stratified by the ABI Type.

#### 3.4.1. Acute Ischemic Stroke and Cardiac Evaluation

In patients with AIS, we observed an increase in the proportion of patients receiving BNP (9.5% in 2014 and 88.8% in 2022, *p* < 0.001), cTnI (12.4% in 2014, 60.1% in 2022, *p* < 0.001), ECG (56.9% in 2014, 66.4% in 2022, *p* < 0.001), and TTE (75.5% in 2014, 83.5% in 2022, *p* < 0.001).

#### 3.4.2. Spontaneous Intracerebral Hemorrhage and Cardiac Evaluation

In patients with sICH, we observed an increase in the proportion of patients receiving BNP (0.9% in 2014, 23.5% in 2022, *p* < 0.001) and cTnI (4.7% in 2014, 58.4% in 2022, *p* < 0.001), with a marginal decline in ECG (78.3% in 2014, 73.8% in 2022, *p* < 0.001) and stable frequency of TTE (39.6% in 2014, 41.7% in 2022, *p* < 0.001).

#### 3.4.3. Spontaneous Subarachnoid Hemorrhage and Cardiac Evaluation

In patients with SAH we observed an increase in the proportion of patients receiving BNP (73.5% in 2014, 80.7% in 2022, *p* < 0.001), cTnI (10.3% in 2014, 90.4% in 2022, *p* < 0.001), ECG (1.5% in 2014, 71.9% in 2022, *p* < 0.001), and TTE (41.2% in 2014, 45.6% in 2022, *p* = 0.0016).

#### 3.4.4. Traumatic Brain Injury and Cardiac Evaluation

In patients with TBI, we observed an increase in the proportion of patients receiving BNP (0% in 2014, 13.6% in 2022, *p* < 0.001), cTnI (15.9% in 2014, 58.4% in 2022, *p* < 0.001), and ECG (71% in 2014, 74.4% in 2022, *p* < 0.001), and a decrease in frequency of TTE (40.8% in 2014, 29.6% in 2022, *p* = 0.0029).

### 3.5. Factors Associated with Cardiac Evaluation in ABI

Table 2 shows the results of multivariable analysis for factors associated with cardiac evaluation across all acute brain injury types.

Overall, 63% (*n* = 7472) received some type of cardiac evaluation. The cardiac evaluation was associated with increasing age, male sex (aOR 1.16 [1.07, 1.27]), non-white ethnicity (aOR 1.11 [1.01, 1.22]), non-commercial insurance (aOR 1.21 [1.09, 1.33]), pre-admission cardiac disorder (aOR 1.21 [1.09, 1.34]), mechanical ventilation (aOR1.78 [1.57, 2.02]), and ICP monitoring (aOR1.68 [1.49, 1.89]). Compared to AIS, sICH (aOR 0.25 [0.22, 0.29]), SAH (aOR 0.36 [0.30, 0.43]), and TBI (aOR 0.19 [0.17, 0.24]) were less likely to receive cardiac evaluation.

### 3.6. Individual Cardiac Testing in ABI

Table 3 provides the results of the multivariable analysis examining factors associated with types of cardiac evaluation. All cardiac evaluation types were strongly associated with age, admitting diagnosis, variability between different evaluation types and sex, cardiac comorbidity, insurance carrier, admitting GCS, mechanical ventilation, and ICP monitoring.

### 3.7. Cardiovascular Test Abnormalities and Outcomes

#### 3.7.1. Prevalence of Cardiovascular Test Abnormalities

Amongst 7263 patients with ECG testing, prolonged QTc was observed in 60.7% (*n* = 2769). Amongst 2650 patients receiving a TTE, we observed EF < 40% in 12.3% (*n* = 326).

#### 3.7.2. Associations between Cardiovascular Test Abnormalities and Outcomes

Table 4 highlights the association between cardiovascular abnormalities and clinical outcomes adjusted for age, sex, race/ethnicity, cardiac comorbidity, admitting diagnosis, admission Glasgow Coma Scale score, mechanical ventilation, and intracranial pressure monitoring.

All-cause in-hospital mortality: patients with cTnI > 75th quartile (aOR 4.38 [1.60–11.9], prolonged QTc (aOR 2.09 [1.75; 2.50]), and ejection fraction <40% (aOR 1.70 [1.29; 2.23]) were more likely to be associated with all-cause in-hospital mortality.

Death by neurologic criteria: patients with troponin 25th–50th quartile (aOR 1.65 [1.10–2.47]), troponin 50th–75th quartile (aOR 1.79 [1.22–2.63]), troponin >75th quartile (aOR 2.18 [1.49–3.17]) BNP 50th–75th quartile (aOR 2.86 [1.28–6.40]), BNP >75th quartile (aOR 4.54 [2.09–9.85]), prolonged QTc (aOR 3.41 [2.28; 5.30]), and ejection fraction <40% (aOR 2.47 [1.07; 5.14]) were more likely to be associated with DNC.

Transition to comfort measures only: patients with troponin 50th–75th quartile (aOR 1.77 [1.14–2.73]), troponin > 75th quartile (aOR 1.81 [1.18–2.78]), and prolonged QTc (aOR 1.71 [1.39; 2.12]) were more likely to be associated with transition to CMO.

Discharged to home: patients with prolonged QTc (aOR 0.66 [0.58; 0.76]) were less likely to be discharged home from the hospital.

### 3.8. Exploring the Association between cTnI, BNP, and EF < 40%

The univariable analysis demonstrated that compared to cTnI < 25th quartile, cTnI > 75th quartile was associated with EF < 40% (OR 2.15 [1.34; 3.45]), and compared to BNP < 25th quartile, BNP > 75th quartile was associated with EF < 40% (OR 6.32 [1.43; 27.95]).

## 4. Discussion

This single-center retrospective study examined the frequency and factors associated with early cardiac evaluation (<72 h) after four types of ABI and the frequency and factors associated with cardiovascular abnormalities and associations with clinical outcomes such as all-cause mortality, DNC, CMO, and discharge to home. The main findings from the study are that (1) 63% of patients with ABI receive some cardiac evaluation, with SAH, sICH, and TBI receiving less frequent testing compared to AIS; (2) amongst patients tested, 60% had prolonged QTc and 10% had EF < 40%; (3) cTnI may be associated with all-cause in-hospital mortality, DNC, and CMO; (4) BNP may be associated with DNC and CMO; (5) Prolonged QTc may be associated with all-cause in-hospital mortality, DNC, CMO, and reduced likelihood of being discharged home; (6) EF < 40% may be associated with all-cause in-hospital mortality and DNC, but not with transition to CMO or discharge home; and (7) only cTnI and BNP values > 75th quartile were associated with EF < 40%.

Our study finds that although 63% of all ABI patients receive cardiac evaluations, patients with SAH, sICH, and TBI receive less frequent testing than AIS patients. Our analysis also suggests that older age, male sex, non-white race, non-commercial insurance, and pre-existing cardiac disorders increase the likelihood of undergoing cardiac evaluation in acute-care settings. Lower admission GCS scores and the absence of mechanical ventilation or ICP monitoring are associated with a decreased likelihood of receiving a cardiac evaluation. In a non-protocolized clinical setting, these findings suggest that neurocritical care clinicians may take a targeted approach to cardiac evaluations, prioritizing patients based on demographic and clinical risk factors and specific ABI subtypes. The increasing rates of cardiac workup in our study may reflect increasing awareness of the importance of cardiac abnormalities in patients with ABI, along with the inclusion of cTnI, BNP, and ECG in our SAH admission order set.

Notably, AIS is associated with the highest rate of cardiac evaluation, while other diagnoses such as sICH, SAH, and TBI are less likely to be related to cardiac evaluation. The finding that the frequency of cardiac evaluations is not homogenous amongst ABI types is hypothesis-generating in that the indication for cardiac testing may differ based on the ABI type. For example, in patients with AIS, cardiac evaluation to evaluate cardioembolic sources of stroke or prevalence of cardiac dysrhythmias may make testing routine for all patients. However, in patients with sICH, testing may depend upon illness severity and the presence of cardiac dysfunction such as hypotension. Similarly, in patients with SAH, the testing frequency may depend upon identifying a hypotensive state that is not the commonly observed admission state. Thus, cTnI, BNP, and TTE may depend on deviation from the 95th percentile of cases that need anti-hypertensive treatment after presumed aneurysmal SAH. In TBI, the testing may rely on cardiac dysfunction and the mechanism of injury, such as falls or syncope. Thus, we hypothesize that ABI types, age, illness severity, and other factors may influence cardiac evaluation rates. In the absence of clinical guidelines and recommendations for cardiac testing outside of the American Heart Association/American Stroke Association recommendations for AIS (cardiac monitoring for at least 24 h) [40] and sICH [41], AIS [42,43,44,45,46], sICH [47,48,49], SAH, and TBI may purely depend on the neurocritical care clinicians’ clinical assessment and judgment and the application of evolving literature in clinical practice.

Age demonstrates a significant association with all four tests, with older age groups exhibiting higher adjusted odds ratios than the reference group (18–44 years). Male sex also shows a modest association with increased adjusted odds ratios across all tests. Cardiac comorbidity and non-commercial insurance display variable associations with different tests. Admission diagnosis and GCS categories reveal significant associations with varying adjusted odds ratios across the tests, particularly notable in diagnoses like AIS and TBI, as well as lower GCS scores. Mechanical ventilation and ICP monitoring exhibit notable associations, with mechanical ventilation displaying consistently higher adjusted odds ratios across all tests compared to the reference. The latter two imply that the sicker patients at elevated risk for cardiac dysfunction are getting tested more frequently.

The rates of cardiac evaluation by the specific type of evaluation are not homogeneous. Overall, TTE numbers have steadily declined over the years. There may be several explanations for these. The evolving presence and proficiency with point-of-care ultrasound (POCUS), which is rapidly becoming standard practice in our critical care unit, maybe one reason TTEs may be ordered with lower frequency over time. The POCUS findings may reassure the clinicians that they may be more likely to order TTEs when they observe either an akinetic or hypokinetic myocardium or reduced EF. This is hypothesis-generating at best and needs further exploration in our cohort and other large multicenter cohorts. Older patients are more likely to have conditions of frailty and atherosclerosis disease, and falls are a commonly associated mechanism for TBI in elders, which can lead to not only cardiovascular abnormalities but also severe acute brain injury conditions. Thus, cardiac workups may be intimately linked to the workup of their TBI to identify preventable and treatable causes.

Our study finds that cTnI may be associated with all-cause in-hospital mortality, DNC, and CMO. BNP may be associated with DNC and CMO. Prolonged QTc may be associated with all-cause in-hospital mortality, DNC, CMO, and reduced likelihood of being discharged home. EF < 40% may be associated with all-cause in-hospital mortality and DNC. Our study demonstrated that cTnI values have prognostic significance depending upon the magnitude of the increase, establishing dose responsiveness. Incremental elevation in cTnI values was significantly associated with adjusted odds of DNC, all-cause in-hospital mortality, and CMO. To put this into clinical context, values of cTnI 10 times or more (>0.3 ng/mL) than normal (0.03 ng/mL) seem to have the most significant correlation. Our findings add to the literature of cTnI being independently associated with increased 7-day mortality and unfavorable discharge disposition in patients with AIS [50,51,52] as well as TBI [20] and amongst neurocritically ill patients in general [53]. Interestingly, in our study, discharge to home was not associated with cTnI values, and we can hypothesize this due to the heterogeneity in the cTnI values amongst the ABI types and the association between cTnI and EF < 40%.

BNP, on the other hand, was only associated with DNC or CMO but not with all-cause mortality or discharge to home. Our study demonstrated that BNP levels have prognostic significance depending upon the magnitude of the increase. Interestingly, a high BNP level was associated with a decreased likelihood of being discharged home, which may be reflective of its role in indicating heart failure or other serious cardiac conditions that compromise patient recovery, but this was not statistically significant. It has been reported that for every one-unit increase in BNP, patients are 3.16 times more likely to have poor Modified Rankin Scale for neurologic disability at discharge [13]. Vrtovec et al. found a relationship between elevated BNP levels and QTc interval prolongation. They discovered that increased BNP levels affected the increased duration of the ventricular action potential, a longer QTc interval on electrocardiogram (ECG), and reduced ejection fraction [54]. In patients with acute subdural hematoma, a BNP of >29.4 pg/mL was an independent predictor of post-operative cerebral infarction and was associated with hematoma volumes [55,56]. BNP is also thought to be a novel predictor of early postoperative seizures in patients with unilateral traumatic subdural hematoma [57]. Prolonged QTc demonstrated a consistent association with mortality (all-cause, DNC, and CMO) and discharge to home. This suggests that a prolonged QTc could be an indicator of critical heart rhythm abnormalities that are associated with poor outcomes, similar to findings by Lee et al. [58]. An EF below 40% is associated with all-cause mortality and DNC but not with CMO and discharge home. The presence of EF < 40% in patients with DNC implies the significance of myocardial dysfunction in patients with DNC, which may have implications for potential organ donation. While we did not follow serial TTE examinations in our patients, who may be potential organ donors, a recent randomized controlled trial found that vasopressor requirements may subside in a proportion of patients within a few days after DNC. The association with transitioning to CMO is less pronounced but suggests some impact, and there is no significant effect on the likelihood of being discharged home, which could mean that while a reduced ejection fraction is linked to poor outcomes, it may not be decisive in discharge planning.

### Clinical Implications

The findings of our study have several clinical implications. Even though there may be shared mechanisms of brain-heart crosstalk across the types of ABI, cardiac evaluation in patients other than AIS patients seems primarily driven by ABI type and illness severity. Since the correlation between cardiac biomarkers and cardiac dysfunction may not be predictable, as demonstrated in the stronger correlations between only the highest values of cTnI, BNP, and EF < 40%, practically, clinicians may choose cardiac dysfunction in the form of hypotension as a clinical trigger to obtain cTnI and or BNP. However, this assumes all patients with EF < 40% may present with absolute hypotension after ABI, which needs further exploration. Similarly, not all hypotension is associated with myocardial injury/dysfunction. Therefore, it is hypothetical if we should even obtain cTnI or BNP data as a screening method in all patients with ABI rather than focus our attention on individualizing testing depending upon patient and disease factors, which our study findings seem to imply. The heterogeneity of cTnI and BNP amongst the ABI types suggests that the ABI type may be insufficient to predict the magnitude of elevation of cTnI or BNP, if any. This was an unexpected finding in our study but requires further exploration.

There were some limitations in this study. Our findings were conducted at a single center, which may limit the generalizability of our results to other settings. Additionally, because this study was observational, we cannot establish causality between the cardiovascular factors and discharge outcomes [59]. In addition, selection bias, measurement bias, and confounding effects of other variables were impossible to rule out; therefore, true effect estimates might be overestimated. There are several strengths to this study. First, our study included a large sample size, providing ample statistical power to detect meaningful associations between cardiovascular test abnormalities and discharge outcomes. This is particularly important given the heterogeneity in etiology and severity of the studied ABIs. Second, the comprehensive data collection allowed for a detailed analysis of multiple cardiovascular factors, such as cTnI, BNP, QTc, and EF. This provides a broader perspective on the relationships between these factors and discharge outcomes in patients with ABI. This comprehensive approach is valuable in identifying specific cardiovascular abnormalities that may serve as useful prognostic indicators and targets for future intervention strategies. Third, our study adjusted for several important confounders, such as age, sex, admission diagnosis, and admission GCS, in the multivariable analysis. This adjustment, in addition to accounting for multiple comparisons with Bonferroni correction, strengthens the validity of the observed associations between cardiovascular factors and discharge outcomes, reducing the likelihood that the findings are due to confounding variables. Lastly, our study population comprised patients with diverse severe acute brain injuries, including AIS, SAH, sICH, and TBI. This diversity enhances the generalizability of our findings to various ABI types. It highlights the potential importance of considering cardiovascular testing abnormalities across different etiologies of ABI.

## 5. Conclusions

This large, single-center study demonstrates that cardiac testing increased over the study period but that there are low rates of cardiac testing in TBI, SAH, and sICH compared to AIS. However, there are strong associations between electrocardiography, biomarkers of cardiac injury and heart failure, and echocardiography findings on clinical outcomes in patients with ABI. The findings need validation in a multicenter cohort, and future studies should aim to elucidate the underlying mechanisms further and explore potential therapeutic interventions to improve outcomes for this patient population. This study may help clinicians with potential prognostic factors and early management strategies to optimize patient outcomes in this challenging patient population.

## Figures and Tables

**Table 1:** The categorization of cTnI and BNP by quartiles used for analysis.

Cardiac Troponin (ng/mL)	Median	<25th Quartile	26–50th Quartile	>50–75th Quartile	>75th Quartile
AIS	0.12	0–0.05	0.06–0.12	0.13–0.60	0.61–69
sICH	0.08	0.03–0.04	0.05–0.08	0.09–0.38	0.39–68
SAH	0.24	0.03–0.06	0.07–0.24	0.25–1.21	1.22–58
TBI	0.07	0.03–0.04	0.05–0.07	0.08–0.28	0.29–20.6
Beta-Natriuretic Peptide (pg/mL)	Median	<25th Quartile	26–50th Quartile	>50–75th Quartile	>75th Quartile
AIS	144.5	3–58.75	58.76–144.5	145–347	348–22460
sICH	152	7–68	69–152	153–356	357–2982
SAH	133	11–58	59–133	134–253	254–5679
TBI	168	12–69	69–168	169–427	428–9480

Abbreviations: AIS: acute ischemic stroke; sICH: spontaneous intracerebral hemorrhage; SAH: spontaneous subarachnoid hemorrhage; TBI: traumatic brain injury.

**Table 2:** Factors associated with obtaining cardiac evaluation tests (results of multivariable analysis).

	Overall	No Cardiac Evaluation	Cardiac Evaluation	aOR
	(*n* = 11,822)	(*n* = 4350)	(*n* = 7472)	[95% CI]
Age in years				
18–44	2406 (20.4%)	1268 (29.1%)	1138 (15.2%)	Reference
45–64	4093 (34.6%)	1432 (32.9%)	2661 (35.6%)	1.74 [1.55, 1.95]
65–79	3520 (29.8%)	1093 (25.1%)	2427 (32.5%)	2.11 [1.88, 2.40]
≥80	1803 (15.3%)	557 (12.8%)	1246 (16.7%)	2.48 [2.14, 2.88]
Sex				
Female	4795 (40.6%)	1716 (39.4%)	3079 (41.2%)	Reference
Male	7027 (59.4%)	2634 (60.6%)	4393 (58.8%)	1.16 [1.07, 1.27]
Race category				
White	9007 (76.2%)	3375 (77.6%)	5632 (75.4%)	Reference
Non-white	2815 (23.8%)	975 (22.4%)	1840 (24.6%)	1.11 [1.01, 1.22]
Insurance				
Commercial	2955 (25.0%)	1244 (28.6%)	1711 (22.9%)	Reference
Non-commercial	8867 (75.0%)	3106 (71.4%)	5761 (77.1%)	1.21 [1.09, 1.33]
Pre-admission cardiac disorder				
No	5562 (47.0%)	2460 (56.6%)	3102 (41.5%)	Reference
Yes	6260 (53.0%)	1890 (43.4%)	4370 (58.5%)	1.21 [1.09, 1.34]
Admitting diagnosis				
Acute ischemic stroke	4272 (36.1%)	784 (18.0%)	3488 (46.7%)	Reference
Spontaneous intracerebral hemorrhage	2569 (21.7%)	1186 (27.3%)	1383 (18.5%)	0.25 [0.22, 0.29]
Subarachnoid hemorrhage	1708 (14.4%)	605 (13.9%)	1103 (14.8%)	0.36 [0.30, 0.43]
Traumatic brain injury	3273 (27.7%)	1775 (40.8%)	1498 (20.0%)	0.19 [0.17, 0.24]
Admission GCS				
13–15	6870 (58.1%)	2710 (62.3%)	4160 (55.7%)	Reference
9–12	1455 (12.3%)	396 (9.1%)	1059 (14.2%)	1.21 [1.05, 1.39]
3–8	3497 (29.6%)	1244 (28.6%)	2253 (30.2%)	0.75 [0.66, 0.80]
Mechanical ventilation				
No	6641 (56.2%)	2728 (62.7%)	3913 (52.4%)	Reference
Yes	5181 (43.8%)	1622 (37.3%)	3559 (47.6%)	1.78 [1.57, 2.02]
Intracranial pressure monitoring				
No	9595 (81.2%)	3742 (86.0%)	5853 (78.3%)	Reference
Yes	2227 (18.8%)	608 (14.0%)	1619 (21.7%)	1.68 [1.49, 1.89]

Note: The model was adjusted for age, admission Glasgow Coma Scale score, sex, cardiac comorbidity, insurance carrier, admitting diagnosis, mechanical ventilation, and intracranial pressure monitoring. Abbreviations: aOR: adjusted odds ratio.

**Table 3:** Factors associated with obtaining an ECG, troponin, BNP, transthoracic echocardiogram, or all four tests in patients with acute brain injury (ABI) (multivariable analysis).

	ECG *n* = 7263	cTnI *n* = 4121	BNP *n* = 2653	TTE *n* = 3980	All Four Tests
	aOR [95% CI]	aOR [95% CI]	aOR [95% CI]	aOR [95% CI]	aOR [95% CI]
Age in years					
18–44	Reference	Reference	Reference	Reference	Reference
45–64	1.32 [1.16; 1.49]	2.17 [1.89; 2.50]	1.81 [1.53; 2.15]	1.74 [1.50; 2.02]	1.76 [1.33, 2.36]
65–79	1.59 [1.39; 1.82]	2.79 [2.40; 3.24]	2.46 [2.06; 2.96]	2.67 [2.28; 3.13]	2.33 [1.74, 3.15]
>80	1.82 [1.55; 2.14]	3.28 [2.77; 3.90]	2.47 [2.00; 3.05]	2.74 [2.28; 3.30]	2.30 [1.64, 3.25]
Male sex	1.17 [1.07; 1.27]	1.13 [1.04; 1.24]	1.06 [0.95; 1.17]	1.11 [1.01; 1.22]	1.14 [0.97, 1.34]
Non-white race	1.01 [0.91; 1.12]	1.12 [1.01; 1.24]	1.10 [0.97; 1.24]	0.94 [0.84; 1.05]	1.02 [0.84, 1.23]
Cardiac comorbidity	1.02 [0.93; 1.11]	1.35 [1.23; 1.48]	0.88 [0.79; 0.98]	1.18 [1.07; 1.30]	0.93 [0.79, 1.10]
Non-commercial insurance	1.14 [1.02; 1.27]	1.12 [1.00; 1.25]	0.92 [0.80; 1.05]	1.09 [0.97; 1.24]	1.07 [0.87, 1.33]
Admitting diagnosis					
Acute ischemic stroke	Reference	Reference	Reference	Reference	Reference
Spontaneous intracerebral hemorrhage	0.73 [0.65; 0.81]	0.59 [0.52; 0.66]	0.13 [0.11; 0.16]	0.16 [0.14; 0.18]	0.2 [0.15, 0.26]
Subarachnoid hemorrhage	0.86 [0.75; 0.98]	1.02 [0.90; 1.17]	0.40 [0.34; 0.46]	0.21 [0.18; 0.25]	0.43 [0.33, 0.55]
Traumatic brain injury	0.57 [0.51; 0.64]	0.52 [0.46; 0.58]	0.06 [0.05; 0.08]	0.13 [0.11; 0.14]	0.16 [0.12, 0.21]
Admission GCS					
GCS 13–15	Reference	Reference	Reference	Reference	Reference
GCS 9–12	1.07 [0.94; 1.21]	1.43 [1.26; 1.62]	1.19 [1.03; 1.37]	1.06 [0.92; 1.21]	1.36 [1.10, 1.68]
GCS 3–8	0.70 [0.61; 0.80]	0.97 [0.84; 1.12]	0.82 [0.69; 0.97]	0.76 [0.66; 0.89]	0.97 [0.76, 1.22]
Mechanical ventilation	2.12 [1.90; 2.37]	1.23 [1.09; 1.37]	0.96 [0.84; 1.10]	1.72 [1.52; 1.95]	1.78 [1.47, 2.16]
Intracranial pressure monitoring	1.52 [1.35; 1.72]	1.55 [1.37; 1.76]	1.04 [0.90; 1.21]	1.01 [0.88; 1.17]	1.24 [0.99, 1.54]

Note: The model was adjusted for age, admission Glasgow Coma Scale score, sex, cardiac comorbidity, insurance carrier, admitting diagnosis, mechanical ventilation, and intracranial pressure monitoring. Abbreviations: aOR: adjusted odds ratio; CI: confidence interval.

**Table 4:** Association between cardiovascular abnormalities and clinical outcomes.

	All-Cause in-Hospital Mortality	Death by Neurologic Criteria	Transition to Comfort Measures Only	Discharged to Home from the Hospital
	*n* = 1961	*n* = 329	*n* = 1426	*n* = 4315
	aOR [95% CI]	aOR [95% CI]	aOR [95% CI]	aOR [95% CI]
Cardiac troponin				
<25th quartile	Reference	Reference	Reference	Reference
25–50th quartile	2.04 [0.62–6.71]	1.65 [1.10–2.47]	1.33 [0.83–2.14]	1.13 [0.82–1.56]
>50th–75th quartile	2.50 [0.83–7.50]	1.79 [1.22–2.63]	1.77 [1.14–2.73]	1.26 [0.93–1.72]
>75th quartile	4.38 [1.60–11.9]	2.18 [1.49–3.17]	1.81 [1.18–2.78]	1.08 [0.79–1.49]
Beta-natriuretic peptide				
<25th quartile	Reference	Reference	Reference	Reference
25–50th quartile	0.52 [0.08–3.36]	1.97 [0.85–4.54]	2.18 [0.99–4.77]	1.21 [0.76–1.92]
>50th–75th quartile	2.98 [0.64–13.74]	2.86 [1.28–6.40]	1.75 [0.79–3.86]	1.35 [0.83–2.21]
>75th quartile	1.73 [0.39–7.66]	4.54 [2.09–9.85]	2.44 [1.13–5.22]	0.91 [0.55–1.51]
Prolonged QTc	2.09 [1.75; 2.50]	3.41 [2.28; 5.30]	1.71 [1.39; 2.12]	0.66 [0.58; 0.76]
Ejection fraction < 40%	1.70 [1.29; 2.23]	2.47 [1.07; 5.14]	1.39 [1.00; 1.91]	0.93 [0.70; 1.23]

The model was adjusted for age, sex, race/ethnicity, cardiac comorbidity, admission diagnosis, admission Glasgow Coma Scale score, mechanical ventilation, and intracranial pressure monitoring. Abbreviations: aOR: adjusted odds ratio; CI: confidence interval.

## Data Availability

Data are unavailable due to privacy or ethical restrictions.
